# Foreign body ingestion mimicking irritable bowel syndrome: a case report

**DOI:** 10.1186/1752-1947-4-244

**Published:** 2010-08-04

**Authors:** Ioannis D Komninos, Ioanna G Tsiligianni

**Affiliations:** 1 Neapoli Primary Health Care Centre, Neapoli, Crete, PO 72400, Greece; 2 Agia Barbara Primary Health Care Centre, Agia Barbara, Heraklion, Crete, PO 70003, Greece

## Abstract

**Introduction:**

Foreign body ingestion is associated with a variety of symptoms and complications, often mimicking various diseases. This case report describes an unusual presentation following foreign body ingestion.

**Case presentation:**

A 56-year-old Greek Caucasian woman presented to a primary care setting, in rural Crete, Greece, with complaints of abdominal pain, cramping and bloating, for the last four months. Alternating constipation and diarrhea was reported. The patient had unknowingly ingested a foreign body that resulted in an irritable bowel syndrome-like presentation.

**Conclusions:**

This case report emphasizes the need for a high index of suspicion from physicians for a wide differential in their approach to abdominal complaints, as well as the importance of an individualized approach to patients in the setting of clinical medicine.

## Introduction

Ingestion of foreign bodies is common primarily in children, psychiatric patients, alcoholics, and denture wearing elderly [1,2]. Selivanov *et al*. reported that, in most cases of foreign body ingestion, the most common foreign bodies ingested were coins, bones, food debris, safety pins, and razor blades [3]. Toothpicks and chicken bones were the most common cause of intestinal perforation [3]. In our study our patient unknowingly ingested a foreign body that had a similar shape and texture to a toothpick.

Foreign body ingestion can present without symptoms [4], and in some cases result in a perforation with gastrointestinal bleeding or an obstruction [1,3,5]. Rarely an abscess or an esophagoaortic fistula occurs [6-8]. The foreign body can be found in any location of the gastrointestinal tract or even out of the gastrointestinal tract through a migration process [1,3,5-12]. Foreign bodies, particularly toothpicks, have been reported to mimic renal colic and Crohn's disease [11,13]. To our knowledge, this is the first report of an irritable bowel syndrome (IBS) manifestation caused by the presence of a foreign body in the intestinal tract.

## Case presentation

A 56-year-old Greek Caucasian woman presented to a primary care setting, in rural Crete, Greece complaining of mild lower abdominal pain, cramping and bloating, during the last four months. The pain was located primarily in the left upper quadrant, and often affected the entire abdomen.

Her symptoms gradually worsened over time, with only temporary relief with defecation. She reported that her bowel habits changed approximately one month after the onset of her abdominal symptoms. Alternating constipation and diarrhea was reported, with diarrhea being more predominant. She also reported a sensation of incomplete bowel emptying. A change in the frequency of bowel movements was also reported. She denied any bleeding, fever, or weight loss. She also denied having any aggravating symptoms such as stress and certain foods over the last few months.

Complete physical examination was within normal limits. Vital signs were also within normal limits. Our patient's medical history included hypertension (treated with an angiotensin II receptor antagonist, telmisartan), hypothyroidism (treated with L-thyroxine) and hypercholesterolemia (treated with atorvastatin), as well as some other minor bowel and gastric disorders that were chronic. There were no concerning associated signs or symptoms such as anemia or weight loss that would have led the family physician to initiate further studies. No abdominal or other surgical operations were reported. The family history for colorectal cancer was negative.

The first impression was that the patient had IBS. General dietary advice according to the National Institute for Health and Clinical Excellence (NICE) guidelines for primary care management of IBS were given (regular meals, avoiding long gaps between eating, adequate and appropriate fluid intake). The patient received also mebeverine hydrochloride 135 mg three times daily for three weeks.

The diagnostic approach included laboratory tests and an abdominal ultrasound control. Laboratory tests results revealed a normal complete blood count, normal erythrocyte sedimentation rate and C-reactive protein, and normal stool studies. Abdominal ultrasound revealed that her gall bladder, biliary tree, pancreas, spleen, and right kidney were all within normal limits. The lower portion of the left kidney was difficult to visual secondary to the presence of a loop of bowel.

**Figure 1:**
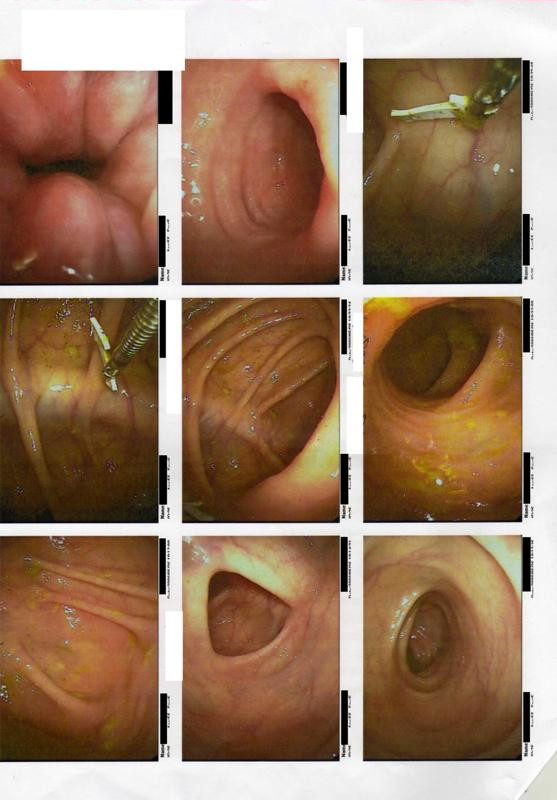
**Colonoscopy images showing the foreign body**.

**Figure 2:**
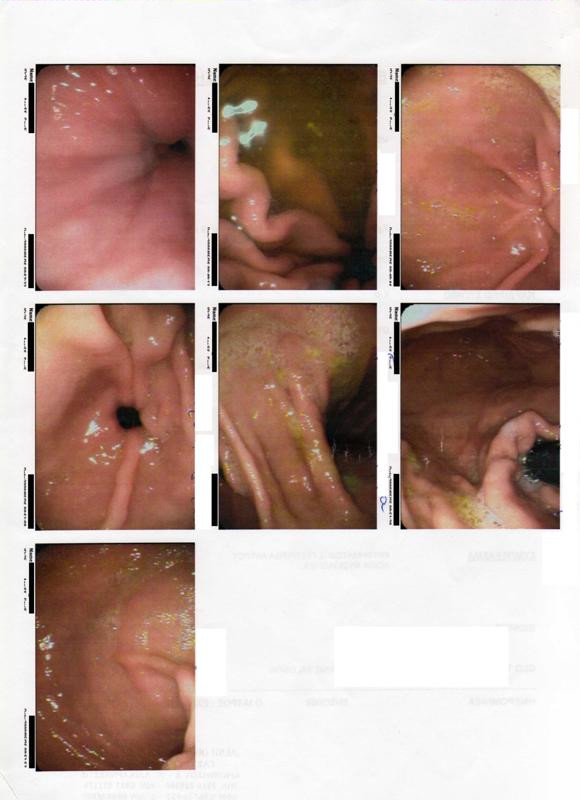
**Colonoscopy images showing increased bowel spasticity**.

**Figure 3:**
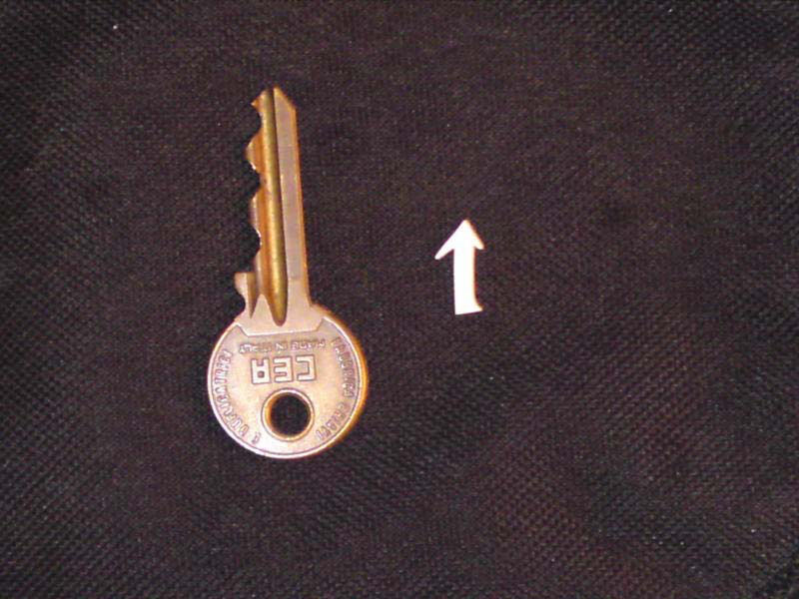
**Dimension of the foreign body in comparison with a key**.

Because her symptoms persisted despite treatment, a colonoscopy was ordered. The colonoscopy revealed the following: rectum with first degree hemorrhoids, sigmoid and descending colon with increased spasticity and normal mucosa, and a normal ileum. In the ascending colon a sharp piece of a birthday cake decoration was found and removed (Figure 1). No necrosis of bowel mucosa or hemorrhage was observed. The increased bowel spasticity that was observed was interpreted by the gastroenterologist who performed the colonoscopy as possibile IBS resulting as a consequence of the foreign body irritation (Figure 2). The dimension of the foreign body is shown in comparison with a key in Figure 3. One week after the removal of the foreign body all symptoms resolved. Our patient was free of symptoms after eight months of follow up.

## Discussion

As reported in one large study of 101 cases of foreign body ingestion, patients are usually examined within 48 hours to six days following ingestion [3]. In our case there was a significant delay because our patient was not aware of the foreign body ingestion, and she attributed her symptoms to her previous history of mild gastrointestinal disorders. In our study the foreign body had the shape and texture similar to a toothpick.

The differential diagnoses considering our patient's age and symptoms included several diseases that would manifest with alternating constipation and diarrhea. These diseases include inflammatory bowel disease, irritable bowel disease, malabsorption syndrome, constipation, medication-induced irritation (anti-emetics, codeine), and mineral deficiencies. In this case report our patient presented with symptoms commonly found in IBS. History, physical examination, and laboratory analysis made the diagnosis of IBS more likely, and excluded other diagnoses.

The Rome III diagnostic criteria for IBS [14,15] are: symptoms lasting at least three months, preceded by at least six months of recurrent abdominal pain or discomfort associated with two or more of the following: improvement with defecation and/or; onset associated with a change in frequency of stool and/or; onset associated with a change in form (appearance) of stool.

Our patient met all three criteria, so an IBS diagnosis was thought most likely. Although the NICE guidelines for the diagnosis and management of IBS in the primary care setting does not indicate ultrasound and/or colonoscopy [16], in our case report we made the decision to individualize our patient's workup based on her unique symptoms and presentation and requested a colonoscopy. The American College of Gastroenterology suggests undergoing a colonoscopy every 10 years, beginning at the age of 50, as the preferred colorectal cancer screening strategy [17]. Taking into consideration our patient's age and the ongoing worsening of her symptoms and pain characteristics, we ordered the colonoscopy that ultimately resolved the diagnostic problem. This case report presentation underlines the importance of an individualized approach to patient care. The fact that after the removal of the foreign body the patient remained free of symptoms in an eight month follow-up suggests that the foreign body was responsible for the IBS-like presentation.

## Limitations

No biopsies from the left and right colon were taken, therefore other etiologies with similar symptoms such as microscopic colitis (lymphocytic, collagenous) could not be excluded.

## Conclusions

This unusual manifestation of IBS-like symptoms as a complication of a foreign body ingestion highlights the need for high index of suspicion from primary care physicians when diagnosing and treating abdominal complaints.

## Consent

Written informed consent was obtained from the patient for publication of this case report and any accompanying images. A copy of the written consent is available for review by the journal's Editor-in-Chief.

## Competing interests

The authors declare that they have no competing interests.

## Authors' contributions

IK, IT analysed and interpreted the patient's data. IT and IK searched the literature for similar cases, and wrote the manuscript. Both authors read and approved the final manuscript.
